# Nausea Predicts Bacteremia in Immunocompetent Patients with Pneumococcal Community-Acquired Pneumonia: Secondary Data Analysis from a Prospective Cohort

**DOI:** 10.3390/jcm12123924

**Published:** 2023-06-08

**Authors:** Hans Kristian Floeystad, Jan Cato Holter, Einar Husebye, William Ward Siljan, Dag Berild, Are Martin Holm, Lars Heggelund

**Affiliations:** 1 Department of Internal Medicine, Sorlandet Hospital, 4615 Kristiansand, Norway; 2 Department of Internal Medicine, Drammen Hospital, Vestre Viken Hospital Trust, 3004 Drammen, Norway; 3 Department of Microbiology, Oslo University Hospital, 0424 Oslo, Norway; 4 Institute of Clinical Medicine, Faculty of Medicine, University of Oslo, 0450 Oslo, Norway; 5 Department of Pulmonary Medicine, Division of Medicine, Akershus University Hospital, 1478 Lørenskog, Norway; 6 Department of Infectious Disease, Oslo University Hospital, 0424 Oslo, Norway; 7 Department of Respiratory Medicine, Oslo University Hospital, 0424 Oslo, Norway; 8 Department of Clinical Science, Bergen Integrated Diagnostic Stewardship Cluster, Faculty of Medicine, University of Bergen, 7804 Bergen, Norway

**Keywords:** nausea, pneumonia, pneumococcal, bacteremia, inflammatory response

## Abstract

Background: In pneumococcal community-acquired pneumonia (CAP), bacteremia is associated with increased mortality, but initial clinical severity scores frequently fail to identify bacteremic patients at risk. We have previously shown that gastrointestinal symptoms are common among patients admitted to the hospital with pneumococcal bacteremia. The aim of this study was to examine gastrointestinal symptoms and inflammatory responses in bacteremic and non-bacteremic pneumococcal CAP in a prospective cohort of immunocompromised and immunocompetent patients hospitalized with CAP. Methods: Logistic regression analysis was used to estimate the predictive value of gastrointestinal symptoms for pneumococcal bacteremia in patients with CAP. The Mann–Whitney test was used to compare inflammatory responses in patients with bacteremic vs. non-bacteremic pneumococcal CAP. Results: Eighty-one patients with pneumococcal CAP were included, of whom 21 (26%) had bacteremia. Immunocompetent patients with pneumococcal CAP had an odds ratio of 16.5 (95% CI 3.0–90.9, *p* = 0.001) for bacteremia if nausea was present, whereas no such association was found in the immunocompromised patients (OR 0.22, 95% CI 0.02–2.05, *p* = 0.18). The serum levels of C-reactive protein, procalcitonin and interleukin 6 were significantly higher in the patients with bacteremic pneumococcal CAP compared to non-bacteremic pneumococcal CAP patients (*p* < 0.001, *p* = 0.005, and *p* = 0.019, respectively). Conclusions: In immunocompetent patients hospitalized with pneumococcal CAP, nausea may be a predictor of bacteremia. Bacteremic pneumococcal CAP patients display an increased inflammatory response compared to non-bacteremic pneumococcal CAP patients.

## 1. Introduction

*Streptococcus pneumoniae* remains the most frequent pathogen in community-acquired pneumonia (CAP), with an estimated prevalence of 20–30% reported for patients with this type of pneumonia requiring hospitalization in Norway. Moreover, 26–33% of the pneumococcal pneumonia patients have bacteremia [1,2].

Previous studies have found several clinical and prognostic differences between bacteremic and non-bacteremic pneumococcal pneumonia [3,4,5]. Host inflammatory responses seem to be higher in bacteremic cases than in non-bacteremic cases [3]. Bacteremic patients are more likely to have complicated features, such as bilateral or multilobar radiological involvement, pleural effusions, septic shock, and treatment failure. Additionally, mechanical ventilation and longer hospital stays are more often required in bacteremic patients [4]. Patients with bacteremic pneumococcal pneumonia have an increased mortality rate compared to non-bacteremic patients, and bacteremia is one of the few predictors of early death in CAP [5].

Several scoring systems predict the risk of death in CAP; CURB-65 [6] and the Pneumonia Severity Index (PSI) [7] are the most commonly used in clinical practice. Since bacteremic pneumococcal infections have poor outcomes, it is reasonable to assume that CAP severity scores may also predict bacteremia. However, studies have reported conflicting results [4,8,9], indicating that although bacteremia is an important prognostic predictor, it is not necessarily recognised by clinical severity scores in CAP patients.

Elevated inflammatory responses in CAP are associated with increased disease severity and mortality rates. However, inflammatory responses are influenced by a number of factors such as symptom duration, immune status of the patient, and patient age [10,11,12,13]. Most importantly, the inflammatory responses seem to be particularly elevated in bacteremic CAP [3,14].

We and others have previously shown an association between gastrointestinal (GI) symptoms and the poor prognosis in bacteremic pneumococcal disease [15,16]. Analogue observations have also been made in other invasive infections, such as group A streptococcal invasive disease [17] and sepsis [18]. Hence, as bacteremic pneumococcal infections may rapidly evolve into septic shock, respiratory failure, and death [4], it is important to investigate the predictive potential of specific GI symptoms.

Whether the specific GI symptoms are different in bacteremic versus non-bacteremic pneumococcal CAP is unknown. In this current study, we explored specific GI symptoms in a well-characterized prospective cohort of hospitalized patients with CAP. The etiology was established through an extensive array of microbiological tests; the cohort included both immunocompetent and immunocompromised patients [1]. We compared GI symptoms and immune responses in cases where *S. pneumoniae* was detected in blood cultures with cases where *S. pneumoniae* was solely detected in a urine antigen test or in an airway specimen by culture and/or quantitative real-time polymerase chain reaction (qPCR). 

The objective of this study was to test the hypothesis that gastrointestinal symptoms are more prevalent in bacteremic than in non-bacteremic pneumococcal CAP patients and that these symptoms are correlated with a more potent inflammatory response.

## 2. Materials and Methods

### 2.1. Study Participants and Design

We performed a secondary analysis of the prevalence of GI symptoms in a prospectively collected, well-defined CAP cohort [1]. All patients were admitted to Drammen Hospital, Vestre Viken Health Trust in South-Eastern Norway, from January 2008 to January 2011. Inclusion criteria have previously been described [1] and included (i) a new pulmonary infiltrate, (ii) fever (rectal temperature > 38.0 °C) and at least one of the following symptoms or signs: cough, dyspnea, respiratory chest pain, crackles or reduced respiratory sounds. All patients were 18 years or older, and patients were excluded if they were discharged from the hospital within 14 days prior to the admission or if they had been hospitalized for more than 48 h.

A total of 320 patients were screened within the first 48 h of admission. Of these, 33 (10%) patients were excluded based on the predefined criteria for the following reasons: previous hospitalization within the past ≤ 2 weeks (2 patients), a chest radiograph was not performed (1 patient), no new infiltrate was detected (19 patients), non-infectious cause of pulmonary infiltrate and/or bronchial obstruction was revealed (7 patients), and fever was not documented (4 patients). A total of 287 patients (90% of the screened population) were eligible for the study. Of these, 4 (1%) patients did not consent to participate in the study. Sixteen patients who entered the study were subsequently withdrawn (6%, 16 of 287 patients) for the following reasons: consent withdrawal (1 patient), previous participation (2 patients), reduced cooperation (2 patients), missing or incorrect ID on case record form (3 patients), inadequate sampling (2 patients), and initial positive chest radiographic findings failed based on a review by a radiologist (6 patients). A total of 267 patients were included in the original cohort.

In the present study, the main analysis cohort was patients diagnosed with pneumococcal CAP. In addition, the prevalence of GI symptoms in pneumococcal CAP was also compared to the non-pneumococcal CAP in the whole CAP cohort [1,19].

### 2.2. Data Collection

The collection of clinical, microbiological and laboratory data have been previously described elsewhere [1,19,20,21]. As the reporting of GI symptoms was not part of the original protocol, each patient’s medical record, including the referral letter and all in-hospital documentation, was reviewed by one of the authors (HKF) to collect this information.

### 2.3. Definitions of Variables

The definitions of the variables have been previously described elsewhere [20,21]. However, some variables were of more importance in this study. The primary outcome was pneumococcal bacteremia. This was defined as the growth of *S. pneumoniae* in the blood culture collected before antibiotic therapy was initiated. A non-bacteremic pneumococcal CAP was defined as, with no growth of *S. pneumoniae* in the blood culture, either positive pneumococcal urinary antigen, or, growth of *S. pneumoniae* in culture from sputum or nasopharyngeal swab, or, pneumococcal DNA corresponding to ≥10^5^ cfu/mL from nasopharyngeal or oropharyngeal samples by use of qPCR [1].

C-reactive protein (CRP), procalcitonin (PCT) and interleukin 6 (IL-6) measured in the patient serum or plasma at hospital admission [19] were used as biomarkers of systemic inflammation.

Patients were classified as displaying GI symptoms if any medical journal document during the first two days of admission noted diarrhoea, abdominal pain, nausea, vomiting or if the patient received antiemetic medication. Reporting of nausea and vomiting or if the patient received antiemetic medication was considered to reflect the same clinical entity and was merged with a single variable denominated nausea.

Immunocompromised patients were defined as those with either primary or acquired immunodeficiency, immunosuppressive medications, haematological malignancy or diabetes mellitus. The primary or acquired immunodeficiency conditions consisted of antibody deficiency, HIV infection, heart, kidney or bone marrow transplant, received chemotherapy within the last 3 months or received radiotherapy within the last 3 months. Immunosuppressive medications included systemic steroids, azathioprine, TNF-α inhibitor, cyclosporine, cyclophosphamide or methotrexate within the previous 3 months.

### 2.4. Statistical Analysis

To evaluate whether GI symptoms may predict bacteremia in pneumococcal CAP, logistic regression analysis with an interaction analysis was performed. Based on the assumption that the predictive value of GI symptoms depended on the level of the inflammatory response, we included immunocompromised patients as an effect modifier in the interaction analysis.

For categorical variables, Pearson’s Chi-squared test was used to evaluate differences between the groups. For the comparison of normally distributed continuous variables between the groups, we used a two-sample t-test, and for the comparison of the non-normally distributed continuous inflammatory variables in different groups, we used the Mann–Whitney test. Tests were considered significant if the two-sided *p* < 0.05.

Data were analysed using STATA/SE, version 16.1.

## 3. Results

Of the originally 267 patients hospitalized with CAP [1], 81 patients (30%) had pneumococcal CAP according to our predefined criteria and were included in the study. Of these, 21 patients (26%) had *S. pneumoniae* growth in blood culture (Table 1).

GI symptoms were present at admission in 12 patients (57%) with bacteremic vs. 23 patients (38%) with non-bacteremic pneumococcal CAP, but the difference was not statistically significant (*p* = 0.13).

**Figure 1:**
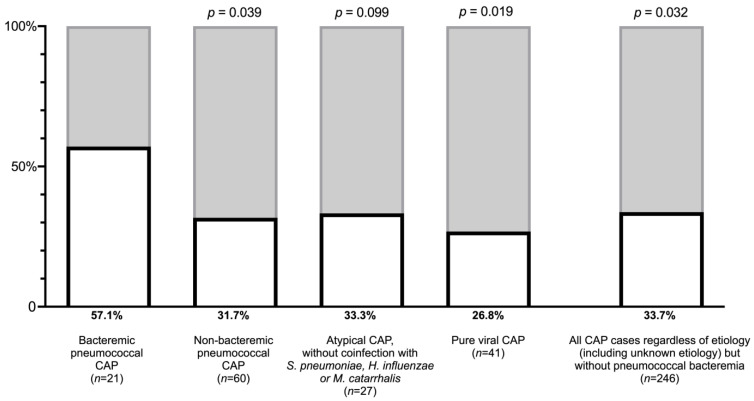
Proportion of patients with nausea at hospital admission for community-acquired pneumonia (CAP) stratified by etiology. *p*-values refer to the comparison between the proportion of nausea among bacteremic pneumococcal CAP and other etiologies by Pearson’s Chi-squared test.

When analysing specific gastrointestinal symptoms separately, a statistically significant higher proportion of bacteremic vs. non-bacteremic pneumococcal CAP cases reported nausea (57% vs. 32%, *p* = 0.039) (Figure 1). However, no statistically significant difference between non-bacteremic and bacteremic pneumococcal CAP patients was observed for diarrhoea (*p* = 0.22) or abdominal pain (*p* = 0.87).

Next, we compared the prevalence of nausea at admission in bacteremic pneumococcal CAP with atypical CAP, pure viral CAP, and all cases of CAP, regardless of microbial etiology but excluding pneumococcal bacteremia, and found that nausea was significantly more prevalent in the bacteremic group, except for when compared to atypical CAP (Figure 1).

In the univariate analysis, we found no statistically significant differences in severe disease defined by a CURB-65 score of >2 in bacteremic versus non-bacteremic pneumococcal CAP patients (*p* = 0.68). However, the statistical power did not allow the CURB-65 score to be included in the multivariate model as a potential confounder for nausea and pneumococcal bacteremia.

The combined short-term outcome of intensive care unit (ICU) admission and/or 30-day mortality was numerically more frequent in bacteremic patients (24%) than in non-bacteremic patients (10%), but this difference was not statistically significant (*p* = 0.11).

**Figure 2:**
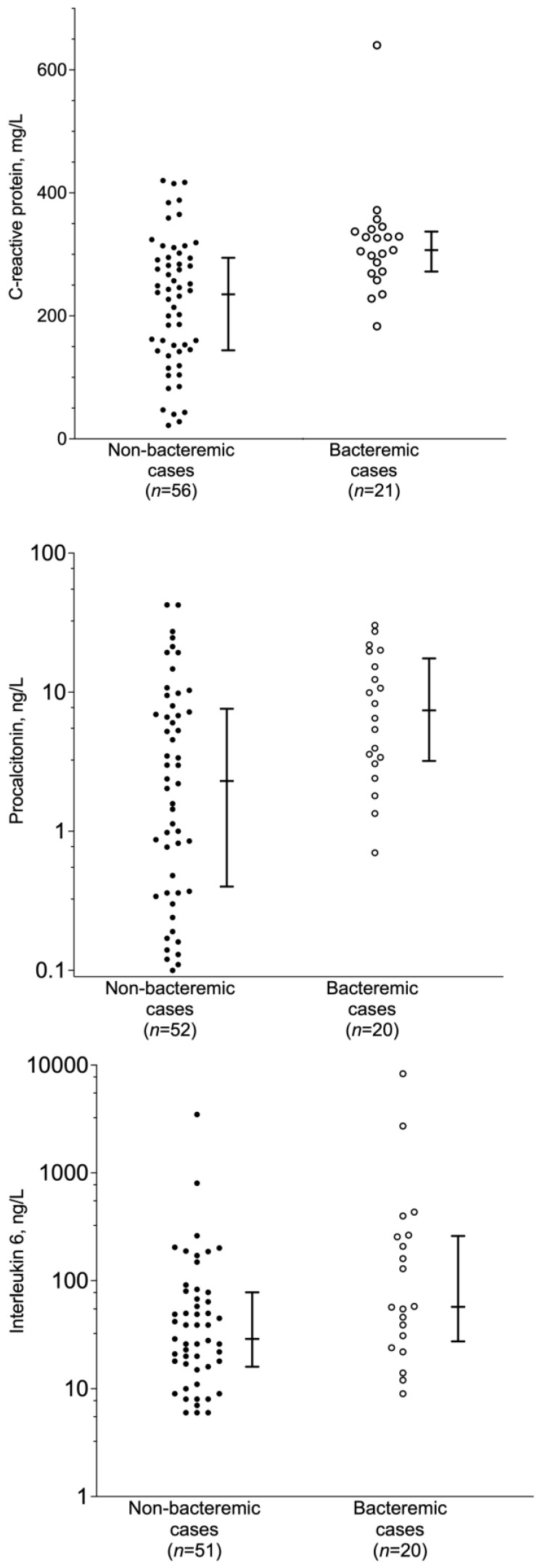
Inflammatory response in bacteremic pneumococcal community-acquired pneumonia (CAP) compared to non-bacteremic pneumococcal CAP, measured by CRP, procalcitonin (PCT) and IL-6 in serum at admission. Data are shown as a scatter plot of individual patient values, median value and interquartile ranges on a linear scale for CRP and on a logarithmic scale for PCT and IL-6. Comparison of CRP, PCT and IL-6 levels for non-bacteremic versus bacteremic pneumococcal CAP by the Mann–Whitney test gave *p* = 0.0004, 0.0049 and 0.0191, respectively.

The inflammatory responses at hospital admission in the bacteremic patients were higher compared to the non-bacteremic patients, with higher levels of CRP, procalcitonin and IL-6 (*p* = 0.0004, *p* = 0.0049 and *p* = 0.0191, respectively) (Figure 2).

**Figure 3:**
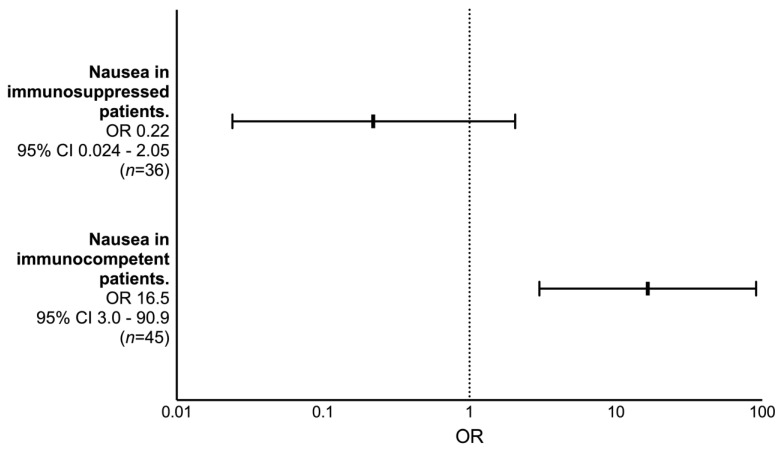
Logistic regression analysis of nausea as a predictor for pneumococcal bacteremia in pneumococcal community-acquired pneumonia, adjusted for immunosuppression as an effect modifier using an interaction term.

In the logistic regression analysis using nausea as a predictor of bacteremia in pneumococcal CAP, we found a statistically significant interaction between nausea and whether the patients were immunocompromised or not (*p* = 0.003). Immunocompetent patients with nausea at admission had an increased odds ratio of 16.5 for pneumococcal bacteremia (*p* = 0.001) (Figure 3). In contrast, we did not find such a predictive power for nausea among immunocompromised patients. In fact, immunocompromised patients with nausea at admission displayed a negative trend for pneumococcal bacteremia, but this was not statistically significant (*p* = 0.18) (Figure 3).

## 4. Discussion

The clinical symptom of nausea was significantly more common among bacteremic than non-bacteremic pneumococcal CAP patients in the present study. Furthermore, nausea is a distinct predictor of bacteremia among immunocompetent patients.

There is a large variation in the proportion of patients with reported nausea among those admitted to the hospital for pneumococcal CAP [22,23]. However, the nausea symptom complex is not uniformly defined. In the large EPIC study from Chicago and Nashville, USA, 36% of hospitalized pneumococcal CAP patients had nausea at admission [22]. In comparison, Hung et al. described gastrointestinal discomfort, which included nausea, vomiting, diarrhoea and abdominal pain in 12% of hospitalized pneumococcal pneumonia patients [23]. To clearly define and embrace all nausea symptoms that we believe are relevant, we considered both direct nausea reporting and vomiting and having received antiemetic medication as signs of nausea. Although there was a relatively high prevalence of nausea in all patients with pneumococcal CAP, it was significantly more frequent among those with bacteremia.

Mortality rates are consistently higher in bacteremic pneumococcal pneumonia compared to non-bacteremic cases [3,4,5,24,25]. However, the most used and best validated clinical CAP severity score, CURB-65, may fail to identify patients at risk of a severe outcome [4,8]. In our study, we did not find any statistically significant differences in CURB-65 scores between bacteremic and non-bacteremic cases. Thus, clinical symptoms of nausea deserve further attention in clinical studies as a potential warning symptom of bacteremia, and increased severity in patients with pneumococcal pneumonia may, therefore, be an important alarming symptom of bacteremia and increased severity. Moreover, the clinical importance may translate into other courses of acute severe infections.

We have less data to investigate why the bacteremic patients displayed an increased prevalence of nausea at admission. In our study, we found that the inflammatory responses, as measured by CRP, procalcitonin and IL-6 in serum, were significantly higher in bacteremic than in non-bacteremic pneumococcal CAP patients. The more pronounced inflammatory responses in bacteremic pneumococcal CAP are in line with the results of previous pneumonia studies [3,26]. Furthermore, since nausea seems to be a strong predictor of bacteremia only among immunocompetent patients, we may speculate that nausea may be directly related to the increased inflammatory response, although our data do not allow for further analysis of this potential association.

Interestingly, nausea was more frequently observed in bacteremic pneumococcal CAP than in other CAP cases, regardless of the etiology. This may be perceived as a further confirmation of the potential role of nausea as a predictor of bacteremic pneumococcal CAP and as a clinically important marker of severity already at admission, several days before the microbiological work-up is available.

In a review summarizing when blood cultures are required in immunocompetent patients, Coburn et al. found fever, chills, the clinician’s impression of the presence of bacteremia, SIRS and Shapiro’s clinical prediction rule to be predictors of bacteremia [27]. Vomiting is a minor criterion in Shapiro’s clinical prediction rule [28], but otherwise, gastrointestinal symptoms were not mentioned in the review. Our findings suggest that nausea, whether treated with antiemetics or not, could be one of the factors providing an “impression of the presence of bacteremia” in the clinician.

Few studies have investigated the symptoms of bacteremia and sepsis in immunocompromised patients. Neutropenic patients have been found to have less prevalent signs of infection, but still produce fever [29]. Several studies have found procalcitonin to predict bacteremia in patients with hematological malignancy [30,31,32], but leukopenic hematological patients with bacteremia and sepsis have rather low procalcitonin levels compared to non-leukopenic patients [33]. On the contrary, immunocompetent patients may have more symptoms and signs of bacteremia because of increased inflammatory responses. Unfortunately, due to the limited data in our study, we could not include inflammatory responses as covariates in the multivariate logistic regression analysis. Nevertheless, and regardless of the reasons, nausea did not predict bacteremia among immunocompromised patients in our study.

### Limitations and Strength

(i) There was a low mortality rate in this cohort as only one of the non-bacteremic patients died (1.8%), and none of the bacteremic patients died. A possible explanation for this could be due to the inclusion criteria that were used in this study. The obligate fever inclusion criterion was intended to establish a stringent, pure and well-characterized CAP cohort. Several studies have reported fever at admission to the hospital to be a protective sign for mortality in bacteremia and severe sepsis [34,35], whereas hypothermia has been described to be a negative prognostic marker in CAP [25]. In fact, afebrile bacteremic CAP patients have been reported to have twice as high a mortality rate as febrile patients [36]. Thus, as we did not include afebrile patients, we may have excluded patients with poor outcomes.

(ii) In addition, the inclusion of patients relied on a low number of clinicians, and there was no recruitment in the evenings, nights or weekends. Patients with reduced ability to provide consent for the study were frequently not included. Thus, the study was prone to selection bias in patients with a lower risk of mortality. Nevertheless, there was a trend towards a more frequent combined outcome of ICU admission and/or 30-day mortality in bacteremic patients compared to non-bacteremic patients in the present study.

(iii) As this was a secondary analysis of a prospective cohort, the information about gastrointestinal symptoms was historical, and a selection or observation bias was possible. It is conceivable that any symptoms, such as nausea, may have been more thoroughly reported in more severely sick patients, or that antiemetic medication may have been more readily administered to more severely ill patients. On the other hand, by not asking patients systematically about GI symptoms, we might not have detected less pronounced GI symptoms, leaving us with clinically relevant cases. Moreover, the number of patients included in our study was relatively low, restricting the possibility to adjust the observations for certain possibly confounding variables. Amending both limitations, the original inclusion of patients was performed prospectively with a thorough registration of data and microbiological evaluation, reducing the risk of bias in our study.

(iv) Our investigation of subgroups of GI symptoms resulted in multiple tests with a risk of type I error. We did not correct this, as it could have prevented us from further examining the potential of nausea to predict bacteremia in pneumococcal CAP, and the risk of type II error as the *p*-value in the following analyses was much lower.

Although this study has some sample size and power limitations, we still believe that our observation of an increased occurrence of nausea among bacteremic pneumococcal CAP patients is interesting and potentially clinically relevant as a warning symptom at hospital admission.

## 5. Conclusions

Our study found that nausea may be a clinical predictor of pneumococcal bacteremia in immunocompetent patients admitted to the hospital with pneumococcal CAP. This observation may be useful when estimating the risk of severe outcomes in this frequent condition, and thus may supplement the current clinical risk scores.

## Figures and Tables

**Table 1:** Characteristics of bacteremic vs. non-bacteremic pneumococcal community-acquired pneumonia (CAP) patients.

Characteristics	Bacteremic Pneumococcal CAP (n = 21)	Non-Bacteremic Pneumococcal CAP (n = 60)	Missing Data
Age, mean (95% CI)	57.3 (49.7–64.8)	63.8 (59.0–68.5)	
Male, n (%)	12 (57.1)	31 (51.7)	
Any comorbidity ^(a)^, n (%)	8 (38.1)	37 (61.7)	
Immunocompromised patients ^(b)^, n (%)	8 (38.1)	28 (46.7)	
Active smoker, n (%)	10 (47.6)	18 (30.5)	1
Pneumococcal vaccine (<10 years ago), n (%)	0	4 (8.7)	21
Viral co-infection, n (%)	8 (38.1)	28 (46.7)	
Symptom duration before admission (days), median (IQR)	4 (2.0–5.0)	4 (2.0–9.0)	
Time to clinical stability ^(c)^ (days), median (IQR)	4 (4.0–4.5)	3 (2.5–4.0)	13
Length of hospital stay (days), median (IQR)	4 (4.0–7.0)	4 (3.0–6.5)	
CURB-65 > 2 at admission, n (%)	7 (33.3)	16 (28.6)	4
ICU admission and/or short-term mortality (<30 days), n (%)	5 (23.8)	6 (10.0)	
Laboratory values at admission to hospital, median (IQR):			
Creatinine (μmol/L)	76 (70–94)	80 (65–95)	1
Urea (mmol/L)	7.1 (5.5–9.0)	6.1 (4.6–8.2)	14
Arterial lactate > 2 mmol/L, n (%)	4 (23.5)	5 (9.8)	13
Bilirubin (μmol/L)	13 (8–18)	14 (8–18)	3
ALAT (U/L)	24 (13–36)	20.5 (12.5–36.5)	4
ALP (U/L)	73 (58–99)	75.5 (59.5–91.5)	4

Abbreviations: CAP, community-acquired pneumonia; CI, confidence interval; IQR, interquartile range; CURB-65, confusion, urea, respiratory rate, blood pressure, age ≥ 65; ICU, intensive care unit. ^(a)^ Comorbidities: COPD, diabetes mellitus, asthma, heart failure, dementia, liver disease, renal disease, haematological cancer or active solid malignancy. ^(b)^ Immunosuppression defining immunocompromised patients: Either primary or acquired immunodeficiency, immunosuppressive medications, haematological malignancy, or diabetes mellitus. Primary or acquired immunodeficiency conditions consisted of antibody deficiency, HIV infection, heart, kidney or bone marrow transplant, received chemotherapy within the last 3 months or received radiotherapy within the last 3 months. Immunosuppressive medications included systemic steroids, azathioprine, TNF-α inhibitor, cyclosporine, cyclophosphamide or methotrexate within the previous 3 months. ^(c)^ Clinical stability was defined as a minimum of three out of four criteria fulfilled: (i) Unchanged antibiotic treatment the last two days, (ii) improvement of general condition, (iii) morning rectal temperature < 38.0 °C, and (iv) >25% decrease in CRP levels or leucocyte cell count.

## Data Availability

The datasets used during the current study are available from the corresponding author upon reasonable request.
